# Risk factors of adjacent segmental fractures when percutaneous vertebroplasty is performed for the treatment of osteoporotic thoracolumbar fractures

**DOI:** 10.1038/s41598-019-57355-1

**Published:** 2020-01-15

**Authors:** Xinjie Liang, Weiyang Zhong, Xiaoji Luo, Zhengxue Quan

**Affiliations:** 1 Department of Pain Management, The First Affiliated Hospital of Chongqing Medical University, Chongqing, China; 2 Department of Orthopaedic Surgery, The First Affiliated Hospital of Chongqing Medical University, Chongqing, China

**Keywords:** Medical imaging, Neurosurgery

## Abstract

The study aimed to analyze the radiographic and magnetic resonance imaging (MRI) findings that might predict the risk for adjacent segmental fractures (ASFs) when percutaneous vertebroplasty (PV) is used for the treatment of osteoporotic thoracolumbar fractures (OTFs). A total of 92 OTFs patients who underwent PV between January 2013 and January 2015 were retrospectively reviewed. The visual analog scale (VAS), Oswestry-Disability Index (ODI) and radiolographic measurements were assessed. The VAS and ODI scores improved significantly at the final follow-up (FU) compared with the preoperation scores. Compared with the preoperative values, the fractured body alignment (FBA) significantly improved at the 3-month FU and the final FU, but the adjacent segment alignment (ASA) and thoracolumbar alignment (TLA) did not improve. According to the correlation analysis, the final FU TLA and the final FU ASA were correlated with the preoperative FBA, ASA, and TLA on plain radiography and were highly correlated on MRI. However, the final FU FBA was not correlated with the preoperative FBA, ASA, or TLA on plain radiography or MRI (P > 0.05). The ASFs were correlated with the 3-month FU TLA (r = 0.6044, P = 0.0037) and the final FU TLA (r = 0.5699, P = 0.007) on plain radiography, and the final TLA was more correlated with the preoperative FBA, ASA, and TLA on MRI than on plain radiography. In conclusion, the preoperative ASA and TLA on MRI were risk factors associated with ASFs in OTFs treated with PV.

## Introduction

Osteoporotic thoracolumbar fractures (OTFs) occur frequently in the elderly population, leading to chronic back pain and progressive sagittal imbalance and even neurological impairment^1–4^. Conservative treatments, including bed rest, nonsteroidal anti-inflammatory drugs, osteoporotic drugs, deep venous thrombosis prevention management and the use of a thoracolumbar brace in the erect position, have been demonstrated for OTFs^5,6^. OTFs can also be managed by percutaneous vertebroplasty (PV) or percutaneous kyphoplasty (PK). Both treatments are not perfect, and although various studies have described the outcomes and complications of each treatment, there is still no consensus on the optimal treatment regimen for OTFs^7,8^. Our study was a retrospective analysis that reported a series of OTF patients treated with PV, and it investigated the risk factors for adjacent segmental fractures (ASFs) based on the assessment of fractured vertebral kyphosis alignment on plain radiography and magnetic resonance imaging (MRI).

## Materials and Methods

### Patient population

This study was approved by the Institutional Review Board of the First Affiliated Hospital of Chongqing Medical University and was conducted according to the principles of the Declaration of Helsinki. All the patients provided their written informed consent to participate in our study prior to the storage of their data in the hospital database. Patients who underwent PV between January 2013 and January 2015 were retrospectively reviewed. Inclusion criteria: only an acute one-level fracture of the thoracolumbar spine from T10 to L2, diagnosed by MRI, the presence of osteoporosis (bone mineral density (BMD) <80 mg/cm^3^ and T score < −2.5), suffered trauma based on low energy, initial treatment with PV and a minimum follow-up (FU) of 2 years. Exclusion criteria: metastatic fractures, primary tumors, high-energy trauma fractures or the presence of neurological impairment, severe compression fractures or severe kyphosis.

### Surgical techniques

PV was performed bilaterally under C-arm X-ray guidance (Ziehm Imaging Systems) using sufficient local anesthesia. Two bone needles were percutaneously inserted into the posterior one-third of the fractured vertebral body. The working cannulas were transpedicularly advanced into the vertebral body. Afterwards, polymethylmethacrylate (PMMA) was slowly injected into the fractured vertebral body. The amount of surgical hemorrhage, the surgical time, the cement volume and complications were recorded accordingly.

### Outcome assessment

**Figure 1:**
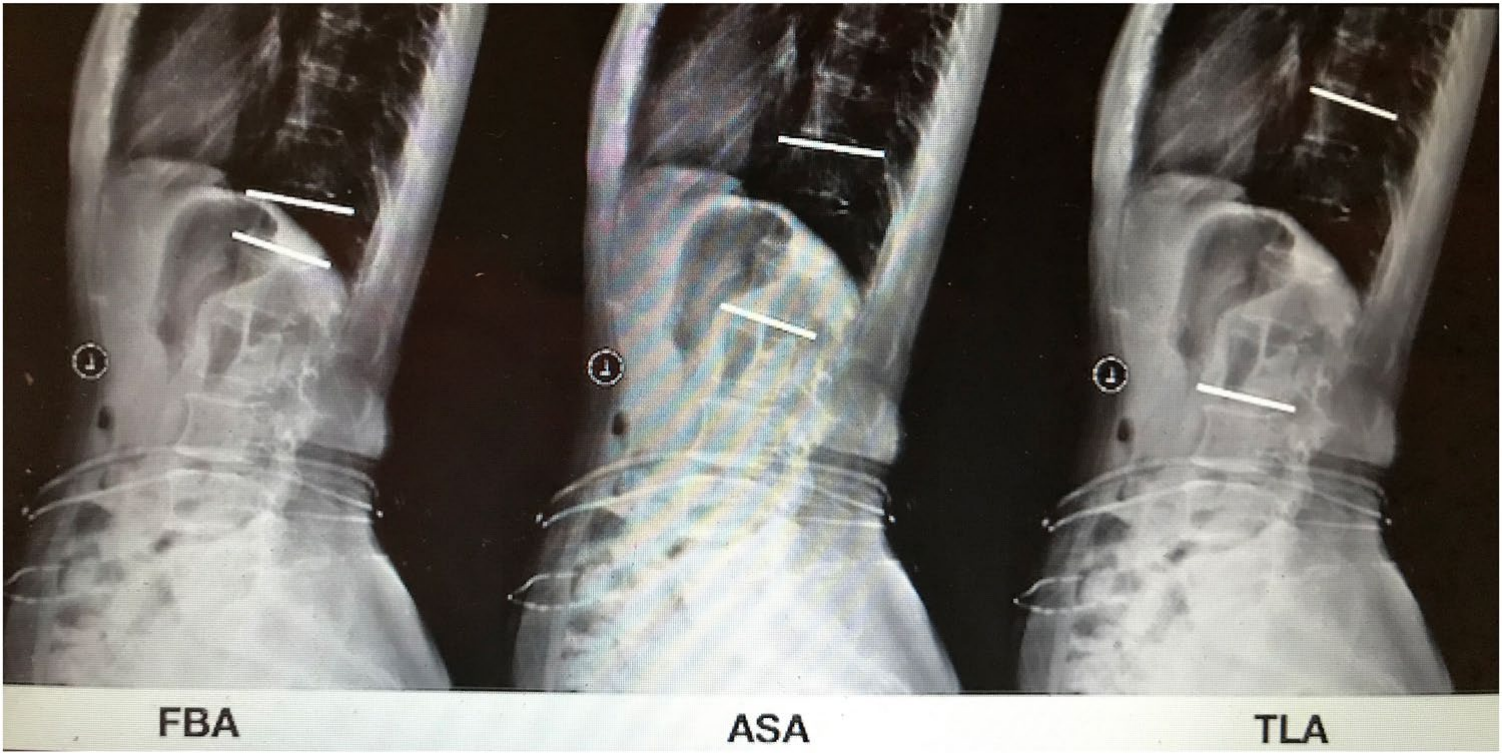
The FBA, ASA and TLA.

In our hospital, plain radiographs were obtained in the standing position, and MRI was obtained in the supine position. The radiographic assessment was performed by two other independent observers (a senior spine surgeon and an attending radiologist who had not participated in the surgeries). For all cases, the following data were observed preoperation, 3 months postoperatively and at the final FU: the (1) operation time, amount of surgical hemorrhage, hospitalization time, BMD, body mass index (BMI), cement leakage, and number of new fractures; (2) visual analog scale (VAS) and Oswestry-Disability Index (ODI); and (3) fractured body alignment (FBA), adjacent segment alignment (ASA) and thoracolumbar alignment (TLA). The FBA was measured using Cobb’s method between the upper end-plate and lower end-plate of the fractured vertebra. The ASA was measured using Cobb’s method between the upper end-plate of the upper vertebra and the lower end-plate of the lower vertebra. The TLA was measured using Cobb’s method between the upper end-plate of T10 and the lower end-plate of L2 (Fig. 1).

### Statistical analysis

The statistical analysis was performed using Student’s t-test, and a correlation analysis was performed using the Statistic Analysis System (SAS Institute Inc., Cary, NC, USA). The results were expressed as the group means ± SDs. Differences with a P-value < 0.05 were considered significant.

## Results

The patients with a mean age of 72.24 ± 9.63 years were followed for an average of 40.30 ± 10.50 months. The VAS and ODI scores significantly improved (VAS t = 13.02, P < 0.0001, ODI t = 35.94, P < 0.0001) at the final FU compared with the preoperation values (Table 1).

**Table 1:** Characteristics and clinical findings.

Parameter	Value
No. of patients (n)	92
Male/female (n)	22/70
Mean age (years)	72.24 ± 9.63
BMD	3.59 ± 0.5
BMI	23.24 ± 2.9
Hospitalization time (d)	13.33 ± 10.17
**Affected level**	
T10	10
T11	5
T12	40
L1	27
L2	10
Surgery time (min)	39.14 ± 14.51
Cement volume (ml)	5.43 ± 1.52
Surgical hemorrhage (ml)	5.71 ± 1.79
**VAS score**	
before treatment	6.24 ± 0.94
final FU	2.23 ± 1.04*
**ODI score**	
before treatment	41.29 ± 3.90
final FU	5.38 ± 2.40*

*Before treatment vs final FU, P < 0.05.

Compared with the preoperative values, the postoperative FBA values significantly improved (3-month FU, t = 10.07, P < 0.0001; final FU t = 7.35, P < 0.0001). However, the ASA (3-month FU, t = 5.956, P < 0.0001; final FU t = 1.167, P = 0.2502) and TLA (3-month FU, t = 0.2825, P = 0.7790; final FU t = 1.096, P = 0.2796) (Table 2) values did not improve significantly. Ten cases of cement leakage and 14 cases of new fractures were observed, neither of which were associated with complications nor required revision surgery.

**Table 2:** The radiographic outcomes.

	X-ray	MRI	P value
**FBA (°)**			
Before treatment	15.37 ± 13.58	8.58 ± 8.14	<0.0001
3 months postop	15.37 ± 13.58*		
Final follow-up	11.73 ± 5.34*		
**ASA(**°**)**			
Before treatment	21.15 ± 17.63	17.68 ± 13.60	<0.0001
3 months postop	17.68 ± 13.60*		
Final follow-up	22.71 ± 14.35		
**TLA(°)**			
Before treatment	24.11 ± 18.60	10.03 ± 2.40	<0.0001
3 months postop	22.52 ± 17.90		
Final follow-up	30.16 ± 13.71		

*Before treatment vs FU, P < 0.05.

**Figure 2:**
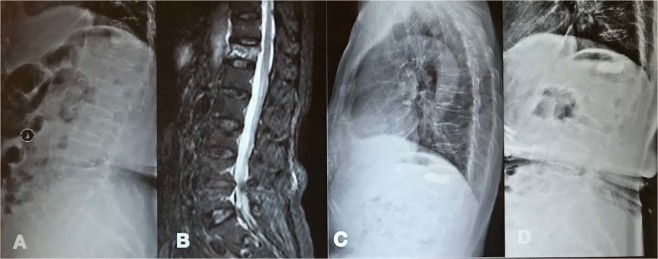
A 61-year-old female patient with an L1 osteoporotic fracture. T12 was treated surgically with PV. (AB) Preoperative X-ray and MRI demonstrated a T12 fracture, and the preoperative ASA and TLA were 29.64° and 25.60° on plain radiography and 8.31° and 15.66° on MRI, respectively. She underwent PV. (CD) At the 3-month FU and 4-year FU, the X-ray showed that the ASA and TLA worsened. L1 fracture was observed.

According to the correlation analysis, the final FU TLA and the final FU ASA were correlated with the preoperative FBA, ASA, and TLA on plain radiography and were also highly correlated on MRI. However, the final FU FBA was not correlated with the preoperative FBA, ASA, and TLA on plain radiography or MRI (P > 0.05) (Table 3). The ASFs were correlated with the 3-month FU TLA (r = 0.6044, P = 0.0037) and the final FU TLA (r = 0.5699, P = 0.007) on plain radiography, and the final TLA was more correlated with the preoperative FBA, ASA, and TLA on MRI than on plain radiography. Therefore, a larger ASA and TLA on MRI could cause severe kyphosis, resulting in significant clinical symptoms, such as back pain, which was more frequently observed in the patients; additionally, even new adjacent fractures or neurological impairment may have occurred (Fig. 2).

**Table 3:** The correlation analysis.

	Final FU FBA	Final FU ASA	Final FU TLA
Preoperative FBA on plain radiography	r = 0.0047 P > 0.05	r = 0.5996 P = 0.0041	r = 0.2111 P = 0.0361
Preoperative ASA on plain radiography	r = 0.1675 P > 0.05	r = 0.3565 P = 0.0043	r = 0.4067 P = 0.0019
Preoperative TLA on plain radiography	r = 0.2067 P > 0.05	r = 0.5895 P = 0.0049	r = 0.4736 P = 0.0006
Preoperative FBA on MRI	r = 0.1262 P > 0.05	r = 0.6811 P = 0.0007	r = 0.2688 P = 0.0161
Preoperative ASA on MRI	r = 0.1591 P > 0.05	r = 0.6774 P = 0.0007	r = 0.3565 P = 0.0043
Preoperative TLA on MRI	r = 0.1996 P > 0.05	r = 0.5676 P = 0.0073	r = 0.5128 P = 0.0003

## Discussion

Compared with those of conservative treatment, the outcomes of PV have been reported to benefit patients, thus providing better pain relief. However, the beneficial effects of PV have been reported to be associated with a higher incidence of new ASFs compared to conservative treatment^1–8^. Studies have reported that PV is not advised for treating OTFs in clinical routine practice, and we are unable to determine whether PV causes new symptomatic vertebral fractures and/or serious adverse events as well as an increased risk of PV in acute OTFs^9–12^. There are no severe complications due to treatment, and although PMMA leakage frequently occurs, it is always asymptomatic^13–15^.

We observed that the radiographic outcomes of PV appeared to provide better vertebral height restoration but worse thoracolumbar kyphosis alignment. In our study, the final FU FBA was not correlated with the preoperative FBA, ASA, or TLA on plain radiography or on MRI (P > 0.05). The final FU ASA and TLA were correlated with the preoperative FBA, ASA and TLA on plain radiography and MRI, and the ASA and TLA were highly correlated on MRI. However, vertebral height restoration could not improve segmental kyphosis or even thoracolumbar kyphosis. Furthermore, the ASFs were correlated with 3-month FU TLA and final FU TLA; we believe that the worse thoracolumbar kyphosis outcome from PV may depend on the significantly higher incidence of new ASFs found in the study^16–19^. Therefore, a higher ASA and TLA, especially on MRI, may be strong risk factors because these vertebrae bear a significant load of compressive forces, leading to progressive adjacent segmental vertebral collapse. The reason for this finding is that in our hospital, plain radiographs are obtained in the standing position and MRI in the supine position. On MRI, sagittal parameters are closer to the actual intraoperative parameters, which are achieved as much as possible during surgery. Hence, preoperative hyperextension training helps restore vertebral height, even the ASA and TLA, which can improve local and global alignment during surgery. However, this position change results in a difference in deformity, which requires the attention of spine surgeons. Whether patients require a unified position for imaging examinations before surgery and which one is more suitable for functional needs or biomechanical needs remains unknown. Future studies should investigate different methods for positioning during imaging examinations in detail.

## Conclusion

The preoperative ASA and TLA on MRI were risk factors associated with ASFs in the treatment of OTFs with PV, which could eliminate complications. However, our study has some limitations. First, the retrospective nature of the small-sample study may be associated with bias. Second, PK patients with OTFs were not included, and only one-level OTFs were discussed. Third, patients with severe compression fractures or severe kyphosis were not included, which may be associated with bias. In the future, prospective, randomized, grouped studies with long-term follow-up periods are needed.
